# Early or Delayed Intervention for Bile Duct Injuries following Laparoscopic Cholecystectomy? A Dilemma Looking for an Answer

**DOI:** 10.1155/2015/104235

**Published:** 2015-02-02

**Authors:** Evangelos Felekouras, Athanasios Petrou, Kyriakos Neofytou, Demetrios Moris, Nikolaos Dimitrokallis, Konstantinos Bramis, John Griniatsos, Emmanouil Pikoulis, Theodoros Diamantis

**Affiliations:** 1st Department of Surgery, University of Athens Medical School, Laikon General Hospital, Agiou Thoma 17 Street, 11527 Athens, Greece

## Abstract

*Background*. To evaluate the effect of timing of management and intervention on outcomes of bile duct injury. *Materials and Methods*. We retrospectively analyzed 92 patients between 1991 and 2011. Data concerned patient's demographic characteristics, type of injury (according to Strasberg classification), time to referral, diagnostic procedures, timing of surgical management, and final outcome. The endpoint was the comparison of postoperative morbidity (stricture, recurrent cholangitis, required interventions/dilations, and redo reconstruction) and mortality between early (less than 2 weeks) and late (over 12 weeks) surgical reconstruction. *Results*. Three patients were treated conservatively, two patients were treated with percutaneous drainage, and 13 patients underwent PTC or ERCP. In total 74 patients were operated on in our unit. 58 of them underwent surgical reconstruction by end-to-side Roux-en-Y hepaticojejunostomy, 11 underwent primary bile duct repair, and the remaining 5 underwent more complex procedures. Of the 56 patients, 34 patients were submitted to early reconstruction, while 22 patients were submitted to late reconstruction. After a median follow-up of 93 months, there were two deaths associated with BDI after LC. Outcomes after early repairs were equal to outcomes after late repairs when performed by specialists. *Conclusions*. Early repair after BDI results in equal outcomes compared with late repair. BDI patients should be referred to centers of expertise and experience.

## 1. Introduction

Since Erich Muhe first described laparoscopic cholecystectomy (LC) in 1985, the treatment of gallstones has dramatically changed, leading to the widespread application of LC among surgeons all over the world. Unfortunately, this application seems to be responsible for the increased rate of complications following the operation, including bile duct injuries (BDI) [1]. Reports have estimated that the incidence of BDI has risen from 0.2–0.4% for open cholecystectomy to 0.6–0.8% for LC, but the true rate still remains unknown [1–4]. There seems to be a trend to more complicated and proximal injuries (injury <2 cm from the bifurcation) [1]. It is known that misinterpretation of anatomy was cited by the majority (92.9%) of surgeons as the primary cause of bile duct injuries whereas 70.9% of surgeons cited a lack of experience as a contributing factor [1].

The management of patients suffering from BDI is a true challenge for every surgeon, particularly for those specialized in hepatobiliary surgery. These patients should always be referred to a tertiary referral center for appropriate treatment due to the complexity of presentation that these injuries tend to have. Cystic duct stump leak, partial laceration of the common bile duct, or even small strictures can be managed by endoscopic retrograde or percutaneous stenting and dilation [5]. The most severe lesions such as bile duct transection or recurrent strictures tend to require reconstructive surgery [5]. Collaboration among surgeons, gastroenterologists, and interventional radiologists is imperative in the management of these complex injuries.

The aim of this study is to record and present our experience in the management of BDI, focusing on early surgical reconstruction and its long-term outcomes.

## 2. Materials and Methods

All patients suffering BDI as a complication of LC managed in the First Department of Surgery, University of Athens Medical School, LAIKO Teaching Hospital, between June 1991 and December 2011 were retrospectively analyzed. Data recorded included patient's demographic characteristics, type of injury according to the Strasberg classification, diagnostic procedures, time of diagnosis, time to referral, type and timing of nonsurgical management, type and timing of surgical management, and final outcome. The follow-up of patients performed by the surgical team that operated on them was also noted.

All patients who were managed for BDI as a complication of LC were identified through the examination of medical notes and computerized data systems. This included both patients who underwent LC at this unit and patients who underwent LC at other units and were later referred to following the diagnosis of BDI. This latter group of patients included all patients referred directly after the diagnosis of BDI and those patients that underwent some type of intervention (surgical or nonsurgical) before the referral.

**Figure 1:**
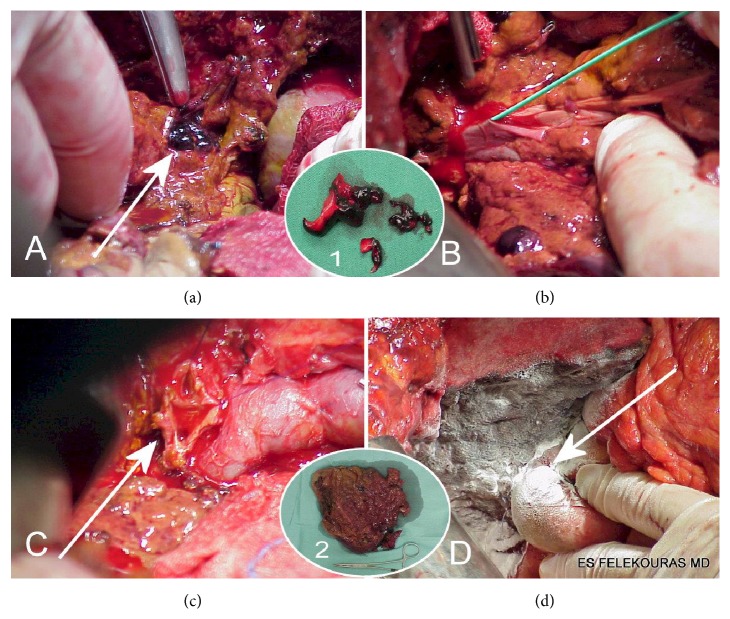
The procedure of right hepatic lobectomy for acute portal and RHA injury following laparoscopic cholecystectomy. (a) The thrombi in the portal vein (white arrow). (b) Thrombectomy of the main truck of the portal vein. (c) The bile duct bifurcation before hepatic lobectomy (white arrow). (d) Right hepatic lobectomy with hepaticojejunostomy on the left (white arrow). Inset 1. The thrombi of the portal vein after thrombectomy. Inset 2. The resected necrotic right hepatic lobe.

As the primary endpoint of the study was the comparison of long-term outcomes between patients who underwent early or late reconstruction of BDI, one patient was excluded from the analysis as he underwent a right hemihepatectomy because of a type E3 injury and a divided and ligated right hepatic artery (RHA) and thrombosed portal vein down to its confluence [6] (Figure 1). All patients gave their informed consent prior to their inclusion in the study.

The presenting symptoms that initially led to the diagnosis of BDI (bile leak, biloma, biliary peritonitis, cholangitis, and obstructive jaundice) were recorded for each patient. Regarding the time of diagnosis, patients were divided into two groups: (1) diagnosis during laparoscopic cholecystectomy; (2) postoperative diagnosis. Time to referral was defined as the time that elapsed between the LC and referral to our unit. This time was defined as zero for patients that underwent LC at this unit. Patients were divided into two groups regarding the time of referral: (1) referral in the first 48 hours after LC (early referral); (2) referral beyond 48 hours after LC (late referral).

The immediate postoperative complications which were evaluated were wound infection, bile leak, biloma, and biliary peritonitis. The long-term postoperative complications which were evaluated were (1) stricture; (2) recurrent cholangitis defined as the occurrence of two episodes of cholangitis in a patient; (3) the need for intervention/dilation defined as the need for any nonsurgical intervention after the surgery (percutaneous drainage of biloma, ERCP and sphincterotomy, and dilation of anastomosis); (4) the need for reoperation.

Initially the patients who were operated on were divided into two groups. The first group consisted of patients who were operated on in other nonspecialized hepatobiliary units with nonspecialized surgeons (non-HBS). The second group was composed of patients who were operated on by specialized hepatobiliary surgeons at this unit. These two groups were compared with regard to the long-term postoperative complications mentioned above.

Patients who underwent surgical repair of BDI by specialized hepatobiliary surgeons at this unit were further divided into two groups according to the time that elapsed since the LC until surgical repair. Patients that underwent surgical repair the first 2 weeks after the LC constituted the first group (early reconstruction group). The late reconstruction group was made up of patients that underwent surgical repair beyond 12 weeks after LC. The endpoint of this study was the comparison of postoperative morbidity (stricture, recurrent cholangitis, required interventions/dilations, and redo reconstruction) and mortality between these two groups (early versus late surgical reconstruction). Further statistical analysis aimed to determine the factors which affected our decision for early versus late surgical intervention.

### 2.1. Operative Technique of Bile Duct Dissection and Roux-en-Y Hepaticojejunostomy Reconstruction after BDI

Extrahepatic biliary tree was explored in all cases up to the confluence of the hepatic ducts (1st or 2nd order) according to the degree of injury. Vital point to the reconstruction was to the finding of a biliary stump(s) that had brisk bleeding cutting edges. If there was not any satisfactory arterial bleeding from the bile duct stumps, the dissection was continued up to the point of bleeding regardless of the level of the reconstruction. This is very crucial to successful hepaticojejunostomy for good early and long-term results [7, 8].

Arterial injuries of the RHA usually do not have to be corrected since the time of repair is early or late so the arteries are already thrombosed. In case of immediate reconstruction of a common or right hepatic artery, injury can be corrected on available expertise (2 cases of ours had immediate reconstruction). If no bleeding of biliary stump was found, a Kasai type bilioenteric anastomosis can be done [9] (1 such case was done and especially in a clockwise fashion which means that the right ischemic ducts were anastomosed first and the left hepatic duct follows) on a patient who did not gave consent for formal right hemihepatectomy which is the first option [10] and liver transplantation the last [11].

**Figure 2:**
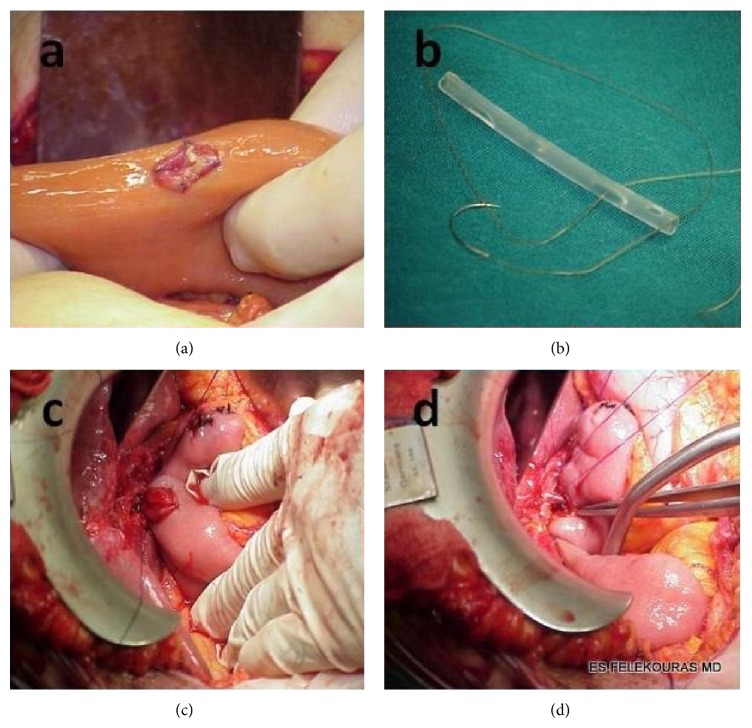
Operative technique of end-to-side Roux-en-Y hepaticojejunostomy. (a) Creation of mucosa-to-mucosa intestinal site of anastomosis. (b) Construction of endoanastomotic (in–in) stent. (c) Hepaticojejunostomy using 4-0 PDS interrupted sutures. (d) Hepaticojejunostomy with endoanastomotic stent.

Then the jejunal loop was transferred to the upper abdomen through the transverse mesocolon on the right side. A single layer hepaticojejunostomy was made between the common hepatic duct and jejunal loop using 4-0 or 5-0 PDS interrupted sutures (Figure 2). For early postoperative protection and improved patency hepaticojejunostomy was stented with an 8–10 Fr Nelaton catheter (Figure 2). The catheter was temporarily secured in place with a single 4-0 absorbable suture. All operations, early and late, were performed by the same surgical team. Drains were placed in all patients.

### 2.2. Statistical Analysis

SPSS statistical software, version 17, was used for data analysis. Common statistics were applied in order to estimate the significance of the results. Chi-square test, Mann-Whitney nonparametric test, and Fischer's exact test were used as appropriate. Differences were considered to be significant if *P* < 0.05.

## 3. Results

There were 42 males and 50 females with a median age of 53 years (range: 33–83 years). Twenty-one (21) injuries occurred in this department, while the remainders were referred from other units. Excluding 22 patients (23.9%) in whom the BDI was recognized during laparoscopic cholecystectomy, the other patients presented with a variety of symptoms after BDI including obstructive jaundice in 21 (22.8%), bile leak in 20 (21.7%), biloma in 13 (14.1%), biliary peritonitis in 5 (5.5%), and cholangitis in 11 (12%) (Table 1). Half the patients had abnormal liver function tests. Patients who were referred early to our institution usually presented with bile leak or biloma or biliary peritonitis, while patients who were referred later presented in most cases with jaundice or episodes of cholangitis.

Diagnostic procedures included magnetic resonance cholangiopancreatography (MRCP), percutaneous transhepatic cholangiography (PTC), endoscopic retrograde cholangiopancreatography (ERCP), transabdominal ultrasound, and abdominal computerized tomography (CT) scans. According to the Strasberg classification of BDI, seven patients (7.6%) suffered type A injury, four (4.3%) type C, eighteen (19.6%) type D, and sixty-three (68.5%) type E (Table 1). We have chosen the Strasberg classification of BDI since it is the most commonly used and gave us the opportunity to design a study that could have comparative results with current literature [12]. In 22 patients (23.9%) the injury was recognized during LC and in 70 (76.1%) during postoperative period. It is worth noting that the frequency of intraoperative recognition of bile duct injuries was much higher in patients who underwent LC in our unit than those who underwent LC in other units (14 patients versus 8 patients, 66.7% versus 11.3%, *P* < 0.001). Time of referral to this institution ranged from the day of BDI to more than three years. Excluding the patients in whom the LC was performed in this department, for the rest of the patients the time of referral was within 48 hours after LC in 26 (early referral) and beyond 48 hours after LC in 46 patients (late referral).

Thirty-five (49.3%) of the 71 patients that were sent to this unit from other hospitals underwent some kind of intervention that is intended to treat the BDI before the referral. Management before referral included percutaneous biliary drainage in seven patients, percutaneous transhepatic or endoscopic biliary stenting in ten patients, and surgical management in 18 patients.

Definitive management of BDI in relation to the type of injury is summarized in Table 2. Conservative management was performed in three patients who after LC had a bile leak which was drained by tube that was placed during the LC. In these patients, bile leak stopped automatically. PTC and ERCP, with or without stent placement, have been the definitive treatment for 13 patients.

Focusing on patients who had undergone surgical repair of BDI, eighteen (18) patients underwent surgical interventions by nonspecialized hepatobiliary surgeons before the referral (Roux-en-Y hepaticojejunostomy in five patients and bile duct repair in thirteen patients) and fifty-six (56) patients underwent surgical interventions by specialized hepatobiliary surgeons in our unit (bile duct repair in three patients, Roux-en-Y hepaticojejunostomy in 48 patients, and more complex procedures in five patients) (Table 3).

In our unit, end-to-side Roux-en-Y hepaticojejunostomy is the procedure of choice for the surgical repair of BDI. Only three of the 56 patients that underwent surgical repair in our unit underwent primary suture of the bile duct and drainage of bile with the placement of a T-tube. These three patients had type D BDI, and the injury was recognized during LC. On the contrary, 72.2% (13/18) of patients who were operated on by non-HBS underwent primary repair of BDI and nine of them had type E injuries. Although these patients are certainly not a representative sample of all patients treated by non-HBS, we can easily attribute the preference of non-HBS in primary repair of BDI to the fact that the end-to-side Roux-en-Y hepaticojejunostomy is a technically demanding surgery, especially for a nonspecialist surgeon (Table 3).

Four patients had concomitant RHA injury (type E in three patients and type D in one patient). Arterial reconstruction was performed in 2 of them in addition to biliary reconstruction. In both cases an end-to-end anastomosis of the RHA following thrombectomy was performed. In the other 2 patients the RHA was not reconstructable. All reconstructed arterial injuries were identified intraoperatively reaching an immediate repair. Since we have chosen Strasberg classification for BDI, vascular injuries were not meticulously evaluated and classified in our study.

Overall long-term morbidity rate of 74 operated patients was 40.5% (30 patients).  Table 4 shows that all the estimated complications (stricture, recurrent cholangitis, the need for intervention/dilation, and the need for reoperation) were more frequent in patients who were operated on by non-HBS. Indeed, comparison between the two groups in the incidence of stricture, need for nonsurgical interventions, or the need for reoperation revealed a statistically significant difference in regard to these complications. Regarding the patients that were operated on by HBS in our department, the overall morbidity was lower (26.8% versus 83.3%, *P* < 0.001).

### 3.1. Early versus Late Reconstruction

Of the 56 patients that were operated on in our unit, 34 were operated on within 2 weeks of the LC (early reconstruction group) and the remaining 22 patients at least 12 weeks after the LC (late reconstruction group).

A total of 13 patients (23.2%) presented with early postoperative complications. Eleven (19.6%) patients presented with wound infection, seven (12.5%) bile leak, five (8.9%) biloma, and one (1.8%) biliary peritonitis. The patient that presented with biliary peritonitis died on the sixth postoperative day due to sepsis and subsequent multiorgan failure. Although patients in the late reconstruction group presented with higher rates of immediate postoperative complications, the statistical analysis revealed no statistically significant differences between the two groups (early versus late reconstruction: wound infection 14.7% versus 27.3%; bile leak 11.8% versus 13.6%; biloma 8.8% versus 9.1%; biliary peritonitis 0% versus 4.5%; overall immediate morbidity 20.6% versus 27.3%) (Table 5).

All patients received follow-up, which ranged from eight to 230 months (median 93 months). During the follow-up period, three patients died from other causes. One patient died seven years after the operation because of secondary biliary cirrhosis caused by stricture of the anastomosis. This patient belonged to the late reconstruction group. He was a 63-year-old man who abandoned the follow-up. He came again to our clinic while he was in end-stage liver failure. The comparison of the overall mortality between the two groups revealed no statistically significant difference (early versus late reconstruction: overall mortality 2.9% versus 4.5%).

During follow-up a total of 15 (26.8%) patients experienced long-term postoperative complications. Eight patients (14.3%) presented with stricture of the anastomosis, four (7.1%) patients presented with recurrent cholangitis, and three (5.6%) patients presented with a combination of these two complications. Both the overall long-term morbidity and individual complications were equal comparing early and late reconstruction groups (early versus late reconstruction: stricture: 18% versus 23%, recurrent cholangitis: 12% versus 14%, need for nonsurgical intervention: 18% versus 23%, and overall morbidity: 24% versus 32%). The long-term complications were managed with intervention with or without dilation and long-term administration of antibiotics. No patient required reoperation (Table 5).

The timing for surgical repair of BDI is influenced by many factors. Although the preferred method for us is early reconstruction, factors such as delay in diagnosis and delay in patient referral to our clinic result in late reconstruction of BDI. Also for patients who underwent LC in others units, underwent no surgical interventions before operation, or suffered biliary peritonitis at the time of the diagnosis it was more likely to operate on late (Table 6).

## 4. Discussion

Bile duct injury remains the most significant and one of the most feared complications after LC that frequently leads to litigation [13, 14]. Many factors lead to this complication, including misinterpretation of anatomy, normal or variant, thermal injury from electrocautery, extensive inflammation, short length of the cystic duct, hemorrhage, and morbid obesity [5, 15–17]. Most of these injuries are not recognized intraoperatively, leading to BDI and consequent increased rates of morbidity and mortality due to severe episodes of cholangitis, jaundice, and intraabdominal sepsis [18–20]. Sometimes the period between injury and definitive treatment is long enough to seriously impact on quality of life. Evidence suggests that these patients have a long history of high rates of admissions to hospitals until their final treatment [21]. Thus early identification and repair can be life saving for patients with bile duct injuries [13].

The final choice of treatment depends upon the type of injury. Usually, when the bile duct has not lost its continuity and the patient does not suffer from severe episodes of cholangitis, more conservative options such as percutaneous drainage or endoscopic stenting are preferred [22]. Alternatively, in cases of complete transection or in the presence of severe symptoms, surgical reconstruction is the treatment of choice. Some cases may even require hepatectomy as the last resort of treatment [6, 23]. Indications for this form of treatment include early (within 5 weeks after LC) vascular injury, proximal BDI, injury to the right hepatic artery, and sepsis caused by liver necrosis or bile duct necrosis [23]. With more chronic patients (over 4 months after LC) hepatectomy effectively manages recurrent cholangitis and liver atrophy [23].

In this institution, Roux-en-Y, end-to-side hepaticojejunostomy is the preferred surgical method [24]. Although the data presented above reveals three patients that underwent primary repair of injured bile duct in this unit, we believe that this type of surgical treatment of BDI should be restricted to patients whose injuries are recognized during LC when these injuries are not type E according to Strasberg calcification. In all the other patients who need surgical management of BDI, we believe that the Roux-en-Y, end-to-side hepaticojejunostomy is the procedure of choice. Five patients suffering injuries of types E4 and E5 underwent more complex procedures (e.g., left bile duct hepaticojejunostomy in combination with modified Kasai procedure for the right biliary tree for a type E5 BDI). Despite long period of study, our surgical approach has not been changed, leading to satisfactory results. The latter is of importance because literature reveals changes in technique to be applied in order to improve operative and long-term results of BDI repair [25].

It must be emphasized that preoperative cholangiography is mandatory in order to obtain an accurate image of the biliary tree. In cases in which the bile duct has been transected, a percutaneous transhepatic cholangiography will correctly predict the anatomic location of injuries in 85% of patients [26]. This is not the case as far as intraoperative cholangiography (IOC) during LC is concerned, because literature is inconclusive or equivocal on this [26–28].

Long-term outcomes in biliary reconstruction are mainly influenced by the level of injury, presence of local inflammation, timing of final repair, type of reconstruction, and experience and expertise of surgeon in these operations and previous attempts of repairs in the same or in other institutions. Patients without history of previous interventions, lack of inflammation, lack of complete transection of common bile duct, and greater diameter of bile duct present better operative results, decreased rates of morbidity and mortality, and lower rates of postoperative complications [29–31].

It is widely accepted that the best results in biliary reconstruction can be achieved in specialized hepatobiliary centers [9, 32–34]. Nevertheless, many general surgeons without previous experience attempt to repair these injuries, often without proper understanding or characterization of the biliary injury. This may be associated with inferior short-term and long-term outcomes, substantial morbidity, and higher rates of complications [18, 35]. Literature suggests that 75% of previous repairs have been attempted by general surgeons without proper experience in BDI [19, 36]. Every failed attempt at repair leads to a decreased bile duct length, making definitive reconstruction more difficult.

Of 71 patients referred to this institution, 18 (25.4%) had a history of previous surgical repair in other units from non-HBS. Although these patients are certainly not a representative sample of all patients treated by non-HBS, the percentage of long-term complications in this subset reached 83.3% in this study. This suggests that all patients, regardless of the type of injury, should be referred to a tertiary high volume center in the early postoperative period.

Final outcome depends on the time of diagnosis and initial treatment and timing of definitive management. The exact time of the operation is a matter of strong debate. Several studies show that patients who undergo operation in the acute phase present with higher rates of perioperative and postoperative complications than patients operated on in a delayed phase [22, 37]. This unit's experience clearly supports the fact that early reconstruction of BDI is as safe as late reconstruction.

Our preference for early reconstruction is demonstrated in patients who underwent LC in our unit (early versus late reconstruction: 87.5% versus 12.5%) or referred to our unit within 48 hours after the LC (early versus late reconstruction: 75% versus 25%). In our unit this approach is followed since 1991 [24]. We believe that this approach reduces both total hospitalization time and the total cost for these patients. Apart from our stated preference for early reconstruction, as illustrated by Table 6, many factors influence the decision for early or late reconstruction. From 56 patients who are operated on in our unit, none is operated on during the intermediate period, two to twelve weeks. We strongly discourage the surgical interventions during this intermediate period because of the inflammation of the biliary tree and surrounding tissues which characterizes the BDI during this period. We must highlight the fact that patients underwent redo operation were initially treated by non-HBS surgeon.

This study builds on the results of other studies which have shown that early repair by a HBS is the superior strategy for the treatment of BDI in properly selected patients regarding outcome, complications, cost, earlier return to normal activity, and quality of life, factors that should be considered in the decisions regarding the management of injured bile ducts [38–40].

Pending a prospective, controlled, randomized trial (evidence level 1) which will show whether an early repair is better than a late one, it is proposed that the operative procedure should be individualized and when conditions allow it should be as soon as possible after the diagnosis of BDI.

## 5. Conclusions

Treatment of patients who suffer from bile duct injury following laparoscopic cholecystectomy is a true challenge for surgeons due to postoperative complications and effect on quality of life. Each patient represents a unique case despite general guidelines that are referred to in the literature and needs detailed investigation before definitive intervention. Patients who undergo early biliary reconstruction after laparoscopic BDI have equal long-term outcomes when the operation is performed in tertiary centers by hepatobiliary specialist surgeons compared to patients who undergo late reconstruction. Delay in referral to a specialist team may contribute to an adverse overall outcome.

## Figures and Tables

**Table 1:** Patients and BDI characteristics.

Age	
Mean (range)	53 (33–83)
Gender, *n* (%)	
Male	42 (45.7)
Female	50 (54.3)
LC performed to, *n* (%)	
Our unit	21 (22.8)
Other units	71 (77.2)
Presenting symptoms, *n* (%)	
Diagnosis during LC	22 (23.9)
Bile leak	20 (21.7)
Biloma	13 (14.1)
Biliary peritonitis	5 (5.5)
Cholangitis	11 (12)
Obstructive jaundice	21 (22.8)
Type of injury according to Strasberg classification, *n* (%)	
Type A	**7 (7.6)**
Type B	**0 (0)**
Type C	**4 (4.3)**
Type D	**18 (19.6)**
Type E	**63 (68.5) **
E1	10 (10.9)
E2	26 (28.3)
E3	22 (23.9)
E4	4 (4.3)
E5	1 (1.1)

**Table 2:** Definite management of BDI according to their type.

Strasberg classification of bile duct injuries (n = 92)			Management					
Type	Description	Number of patients (%)	Conservative (wait and see)	Drainage	PTC	ERCP	Bile duct repair	Reconstruction
Type A	Bile leak from cystic duct stump or the gallbladder bed	7	2	1	1	3	0	0
Type B	Right segmental duct division where both ends are clipped	0	0	0	0	0	0	0
Type C	Right segmental duct division where the hepatic end remains open	4	1	1	2	0	0	0
Type D	Lateral wall injury to the common bile duct	18	0	0	3	4	7	4
Type E	Major CBD division/stricture with 5 subdivisions	**63**	**0**	**0**	**0**	**0**	**9**	**54**
E1	Site of CBD division is >2 cm from the bifurcation	10	0	0	0	0	4	6
E2	Site of CBD division is <2 cm from the bifurcation	26	0	0	0	0	3	23
E3	Site of CBD division is at the bifurcation	22	0	0	0	0	2	20
E4	Division or injury to the left, right, or both hepatic ducts	4	0	0	0	0	0	4
E5	An injury of a right segmental duct along with a type E3/E4 injury	1	0	0	0	0	0	1

Total		92	3	2	6	7	16	58

**Table 3:** Surgical management of bile duct injuries (*n* = 67).

	Total		Bile duct repair		Reconstruction	
	Patients operated on by HBS (%)	Patients operated on by non-HBS (%)	Patients operated on by HBS (%)	Patients operated on by non-HBS (%)	Patients operated on by HBS (%)	Patients operated on by non-HBS (%)
Early (*<*2 weeks) repair or reconstruction	32 (57.1)	7 (38.9)	3 (100)	6 (46.2)	29 (54.7)	1 (20)
Intermediate (2–12 weeks) repair or reconstruction	0 (0)	11 (61.1)	0 (0)	7 (53.8)	0 (0)	4 (80)
Late (*>*12 weeks) repair or reconstruction	24 (42.9)	0 (0)	0 (0)	0 (0)	24 (45.3)	0 (0)

Total	56 (100)	18 (100)	3 (100)	13 (100)	53 (100)	5 (100)

HBS: specialized hepatobiliary surgeons.

Non-HBS: nonspecialized hepatobiliary surgeons.

**Table 4:** Summary of long-term outcomes after surgical intervention to BDI; results by surgeon group.

	Non-HBS (18)	HBS (56)	Total (74)	Significance
Stricture, number (%)	11 (61.1)	11 (19.6%)	22 (29.7)	**0.001**
Recurrent cholangitis, number (%)	4 (22.2)	7 (12.5%)	11 (14.9)	0.445
Intervention/dilation, number (%)	10 (55.6)	11 (19.6%)	21 (28.4)	**0.003**
Redo reconstruction, number (%)	5 (27.8)	0 (0%)	5 (6.8)	**0.001**
Overall long-term morbidity, number (%)	15 (83.3)	15 (26.8%)	30 (40.5)	**<0.001**

**Table 5:** Results of biliary reconstruction by HBS.

	Early (<2 weeks) repair or reconstruction (34)	Late (>12 weeks) repair or reconstruction (22)	Significance	Total (56)
Immediate postoperative complications				
Wound infection, number (%)	5 (14.7)	6 (27.3)	0.310	11 (19.6)
Bile leak, number (%)	4 (11.8)	3 (13.6)	0.999	7 (12.5)
Biloma, number (%)	3 (8.8)	2 (9.1)	0.999	5 (8.9)
Biliary peritonitis, number (%)	0 (0)	1 (4.5)	0.393	1 (1.8)
Overall immediate morbidity, number (%)	7 (20.6)	6 (27.3)	0.563	13 (23.2)
Long-term postoperative complications				
Stricture, number (%)	6 (17.6)	5 (22.72)	0.736	11 (19.6)
Recurrent cholangitis, number (%)	4 (11.8)	3 (13.6)	0.999	7 (12.5)
Intervention/dilation, number (%)	6 (17.6)	5 (22.72)	0.736	11 (19.6)
Redo reconstruction, number (%)	0 (0)	0 (0)	∗	0 (0)
Overall long-term morbidity, number (%)	8 (23.5)	7 (31.8)	0.494	15 (26.8)
Mortality, number (%)	1 (2.9)	1 (4.5)	0.999	2 (3.6)

^*^No statistics are computed because the absence of need of redo reconstruction is a constant.

**Table 6:** Factors that potentially influenced the decision for early or late surgical intervention by HBS.

	Early	Late	*P* value	Total
Injury type				
E (49)	29	20	0.692	49
Non-E (7)	5	2		7
E 1, 2	16	9		25
E 3, 4, 5	13	11	0.484	24
Initial recognition of injury				
During LC	12	2	**0.027**	14
Postoperatively	22	20		42
LC performed to				
Our unit	14	2	**0.009**	16
Other units	20	20		40
Time to referral				
<48 hours	9	3	**0.038**	12
>48 hours	11	17		28
Presenting symptoms				
Bile leak	9	3	0.063	12
Others	13	17		30
Biloma	3	4	0.580	7
Others	19	16		35
Biliary peritonitis	0	5	**0.018**	5
Others	22	15		37
Cholangitis	1	4	0.174	5
Others	21	16		37
Obstructive jaundice	9	4	0.143	13
Others	13	16		29
Nonsurgical interventions before operation				
Yes	7	14	**0.001**	21
No	27	8		35
